# Cooperation between Referees and Authors Increases Peer Review Accuracy

**DOI:** 10.1371/journal.pone.0026895

**Published:** 2011-11-09

**Authors:** Jeffrey T. Leek, Margaret A. Taub, Fernando J. Pineda

**Affiliations:** 1 Department of Biostatistics, Johns Hopkins Bloomberg School of Public Health, Baltimore, Maryland, United States of America; 2 Department of Molecular Microbiology and Immunology, Johns Hopkins Bloomberg School of Public Health, Baltimore, Maryland, United States of America; Hungarian Academy of Sciences, Hungary

## Abstract

Peer review is fundamentally a cooperative process between scientists in a community who agree to review each other's work in an unbiased fashion. Peer review is the foundation for decisions concerning publication in journals, awarding of grants, and academic promotion. Here we perform a laboratory study of open and closed peer review based on an online game. We show that when reviewer behavior was made public under open review, reviewers were rewarded for refereeing and formed significantly more cooperative interactions (13% increase in cooperation, P = 0.018). We also show that referees and authors who participated in cooperative interactions had an 11% higher reviewing accuracy rate (P = 0.016). Our results suggest that increasing cooperation in the peer review process can lead to a decreased risk of reviewing errors.

## Introduction

Peer review is the foundation for decisions concerning publication in journals, awarding of grants, and academic promotion. Anonymous peer review plays a major role in decisions concerning publication in journals, awarding of grants and academic promotion. However, the anonymous nature of peer review is increasingly under scrutiny [1]–[4], and some journals have considered or already moved to open peer review [3], [5]–[7]. Debates about the utility and ethics of anonymity, have led to questions concerning whether there is any science behind peer review [8], to calls for an evidenced-based rationale for peer review [9], and to debates about alternative practices of peer review [2]–[4].

Despite its central role in the scientific process, the underlying social dynamics and accuracy of peer review under alternative systems are difficult to study. It is perhaps not surprising that there are few reliable studies of peer review. Conclusive randomized controlled studies require cooperation and coordination of journals, editors and authors within an academic community. It has been argued that many studies are inconclusive or suffer from methodological defects, primarily due to the robustness of author or review blinding [10]. Moreover, these studies focus on review quality [11]–[16], rather than correctness or impact of the results which can only be assessed retrospectively and after scientific consensus is achieved.

Here we develop a theoretical model for peer-review which can be described in terms of payoffs for author and referee behavior. We analyze the theoretical model to determine the properties of optimal strategies under both open and closed peer review. We then develop a model system in the form of an online game launched from the Amazon EC2 cloud to collect data to both support our theoretical model and evaluate accuracy and social dynamics under peer review. Using our model system, we perform experiments to collect quantitative data about the social behavior of referees in anonymous (closed) and non-anonymous (open) peer review. These data represent the first direct quantitative measurements of peer review accuracy under alternative peer reviewing systems. Using these data we show that: (1) under open review peer reviewers are rewarded for refereeing in contrast to closed review, (2) reviewers and authors are significantly more likely to cooperate under open review versus closed review, and (3) cooperative peer reviewing behavior leads to higher review accuracy.

## Results

### Theoretical Model

#### Definition of the Peer Review Game

In our model there are 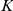 players participating in a game for a total of 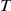 units of time. Each player in the game participates in two activities: (1) solving problems and (2) reviewing solutions of their peers. For player 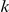, the total time spent reviewing 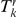 and solving 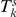 must be less than the total time allocated for playing the game 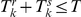. Over the course of the game, player 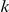 submits 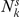 solutions and reviews 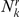 solutions for other players. Let 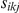 indicate the 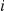
th solution for player 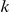, which is reviewed by player 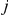. For each solution there is a corresponding time the solution was submitted 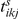 and time that the reviewer completed the review 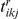. For player 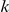 the number of accepted papers at time 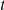 is the sum of the indicators that each of their submitted solutions is accepted up to that point: 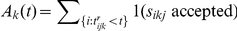.

The payoff is proportional to the number of accepted solutions, which reflects the commonly held belief of “publish or perish” in academia. So the expected payoff for player 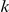 at time 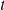 is:
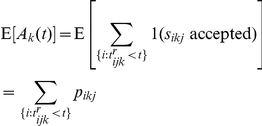
where 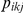 is the probability that solution 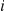 for player 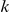 is accepted by player 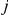. The payoff is a function of the number of submitted solutions and the probability that each solution is accepted. The probability a solution is accepted is a function of the submitter, the reviewer, the time the solution is reviewed, and the solution itself.
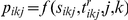
where 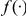 is a non-negative function mapping the solution, the review time, the solver, and the reviewer onto 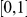. Player 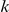 can increase their payoff by increasing the number of solutions they submit or increasing the probability each solution is accepted.

An alternative is a competitive payoff where the payoff function is proportional to the difference between a player's number of accepted solutions and the maximum of all the other player's payoffs. In this case, the expected payoff is:
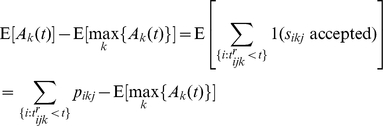



#### Closed Peer Review (CPR)

Under closed peer review, the model for the acceptance probability for solution 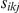 is modeled as:
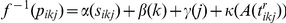
Here there is an effect for the solution itself 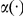 which may reflect a large number of factors about the solution, including the type of problem or the time spent on the solution. There is also an effect for the solver 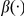 since some solvers are more likely to submit correct solutions than others. Each reviewer may choose to accept or reject problems at a different rate which we model by 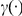. Under CPR the public information is the number of solutions that each player has submitted and had accepted by another player. 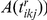 is a vector of the cumulative number of accepted solutions for each player at time 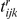. The function 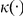 quantifies the influence of this information on the probability solution 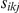 is accepted.

At any given time point a player can choose between three different strategies: (1) solve and submit a problem, (2) review a problem and reject, or (3) review a problem and accept. The first strategy has the potential to improve a player's payoff, by increasing the number of submitted solutions. If a player chooses either of the first two strategies, no other player's score will increase. If the player chooses strategy (3), then another player's score will increase. However, that person will not know who accepted their solution. Under CPR, if a player chooses strategy (2) or (3) they will reduce the amount of time they spend solving a problem and will reduce their expected payoff. However, no other player will be aware of this choice since reviews are anonymous and only the cumulative accepted solutions for each player is known. In this game, there is no increase to the payoff function for reviewing. Therefore, each player maximizes their expected payoff by always choosing strategy (1) and never reviewing, so this solution is the Nash equilibrium [17].

#### Open Peer Review (OPR)

Under OPR the model for the acceptance probability for solution 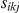 includes the same terms as CPR, along with terms that encode the influence of the current public and private information available to each player.
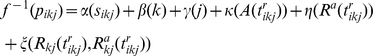
The model includes a term, 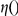, that is a function of vector of the cumulative number of solutions reviewed and accepted by each player. The functions 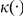 and 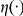 encode the public information available to each player. Under the open system, player 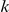 also knows the cumulative number of times player 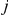 has reviewed their solutions, 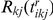, and accepted their solutions 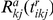 at the time of the review. The function 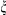 quantifies the effect of this information on the probability of acceptance.

Under the OPR it is possible that a player may incur some benefit by reviewing for other players. Specifically if a player has previously accepted solutions for player 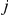, they may improve the probability their solution is accepted through the function 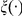. Similarly, if they are a generous reviewer to all the other players, player 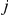 may again be more sympathetic and the probability of acceptance may be increased through the function 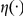. The residual benefit of reviewing may carry over to future times, so the functions 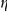 and 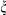 are functions of the cumulative reviews and acceptances to time point 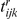.

Under OPR, a player still has the same three strategy choices at any given time point: (1) solve and submit a problem, (2) review a problem and reject, or (3) review a problem and accept. However, under OPR a player may incur some increase in their probability of acceptance if they choose strategy (2) or (3). They are particularly likely to incur increases in their acceptance probability when choosing strategy (3). Under this mode, additional Nash equilibria may be possible. To calculate these equilibria, substantial additional assumptions are required about the benefit of reviewing, the time it costs to perform a review, and the timing of additional reviews. Since the payoffs functions now depend continuously on the number of accepted and reviewed at each time point, the game must be modeled as a continuous game. Theoretical analysis of OPR represents a potentially fruitful area for future research.

#### Relative Payoff of Reviewing and Solving

It is not difficult to argue that in science, the payoff for solving problems is significantly greater than the payoff of reviewing submissions. The only way to change this ordering is to decrease the payoff for solving problems or to increase the payoff for reviewing problems, or both. The former might be achieved in situations where the information available to the community causes the community to punish a player by reducing the acceptance rate of the player's submissions [13]. The latter might be achieved by increasing the time spent reviewing and rejecting the submissions of other players. An example would be if a player could somehow reject all the submissions of a strong competitor, without knowledge of these actions being provided to the community.

### Experimental Results

#### Setup

Our model system for peer review was an online game launched from the Amazon EC2 cloud played by 7–10 individuals over a fixed period. Players were graduate students, postdoctoral fellows, research scientists or principal investigators, all of whom are members of a single research laboratory. The game was designed to replicate several components of editorial peer review: (1) most reviewers know the authors of the papers they referee, (2) peer review is usually performed within relatively small communities of individuals [18], and (3) peer review involves repeated interactions between referees and authors. The game's interface presented players with multiple-choice questions similar to those found on the Graduate Record Exam (GRE) [19]. At any point in the game a player chose between solving problems or reviewing (accepting or rejecting) solutions submitted by other players. The software also played the role of journal editor and randomly assigned submitted solutions to players for review. At the end of the game, the two players with the largest number of accepted submissions received monetary rewards, reflecting the conventional publish or perish academic incentives.

**Figure 1:**
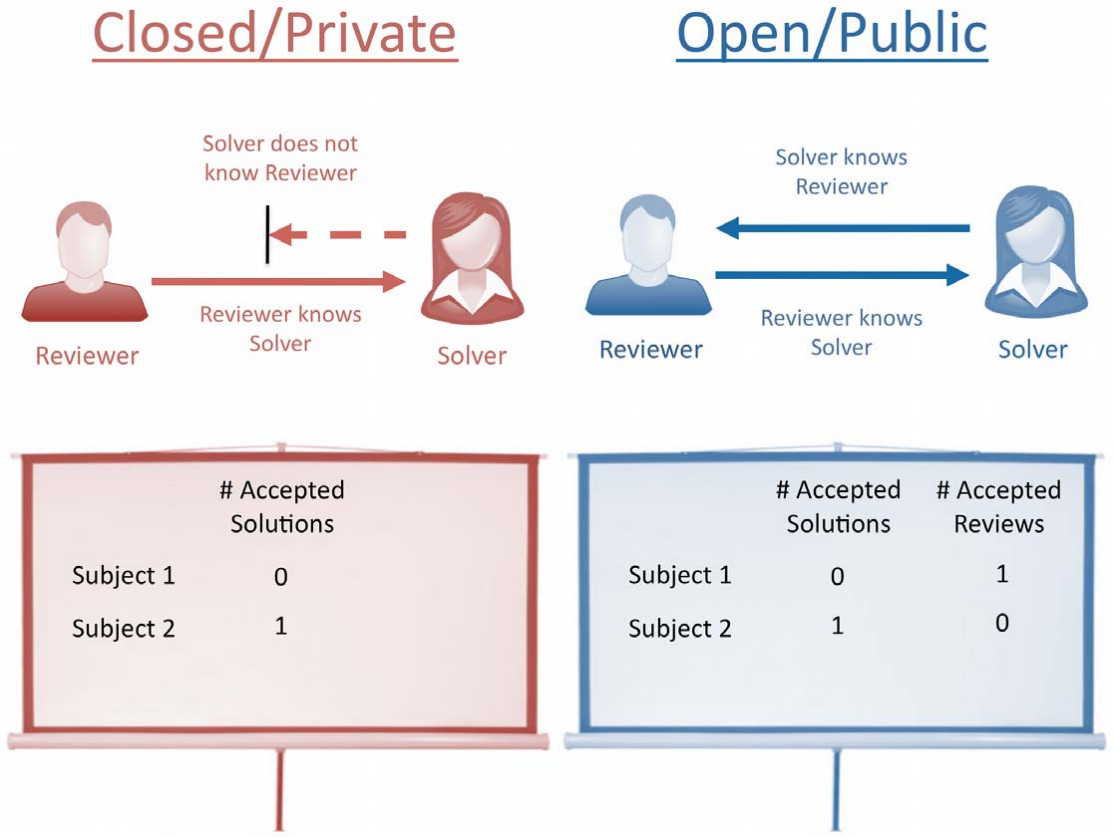
Open versus closed peer review systems for the peer review game. Under the closed system of peer review (left column), reviewers know the identity of problem solvers, but problem solvers do not know the identity of the reviewers. Public information is limited to the number of accepted solutions for each player. Under the open system of peer review (right column) solvers and reviewers are known to each other, and both the number of accepted solutions and accepted reviews for each player are known publicly.

Individual games were played in either a closed mode, or in an open mode. In the closed mode, the reviewers were anonymous (Figure 1 left column). In the open mode, players knew which reviewer accepted or rejected each of their submissions (Figure 1 right column). The public information under the closed mode was the number of submitted solutions that were accepted. In the open mode, both the number of submitted solutions that were accepted and the number of times each player accepted a peers solution were public.

#### Experimental results agree with theoretical model and previous studies of peer review

To mimic the dynamics of a small community of scientists, we recruited individual research laboratories to play the Peer Review Game (Materials and Methods). We recruited members of six research laboratories at Johns Hopkins University to play the Peer Review Game in closed mode (3 labs, n = 8, 8, and 9 players) and open mode (3 labs, n = 7,10, and 8 players). Each laboratory played the game for T = 40 minutes. We collected a total of 1,143 solutions and 666 reviews over the course of the six experiments. Overall, 62% of the submitted solutions were correct. Peer review did lead to an increase in accuracy; only 39% of rejected solutions were correct, while 78% of accepted solutions were correct. We first evaluated our experimental model by comparing our results to predictions of our theoretical model, previous results on iterated games, and previous studies of peer review.

In the open system each solution a player accepted led to an increased probability their own next submission would be accepted (2% increase per accepted solution, P = 0.047). Our theoretical analysis suggested a similar potential increase in probability for helpful reviewing behavior. Under closed review players were not rewarded for reviewing additional submissions, i.e. there was no significant difference in the probability a playerÕs submissions would be accepted for each additional review (0.8% decrease per accepted solution, P = 0.30).

Under the open system one of the top two reviewers was always one of the winners of the game, suggesting that reviewers were rewarded for their good behavior toward other players (Materials and Methods). This result agrees with both our theoretical analysis and the results of previous studies of iterated games, which showed that costly punishment has been shown to be negatively associated with payoff. In other words “winners don't punish” [20].

**Figure 2:**
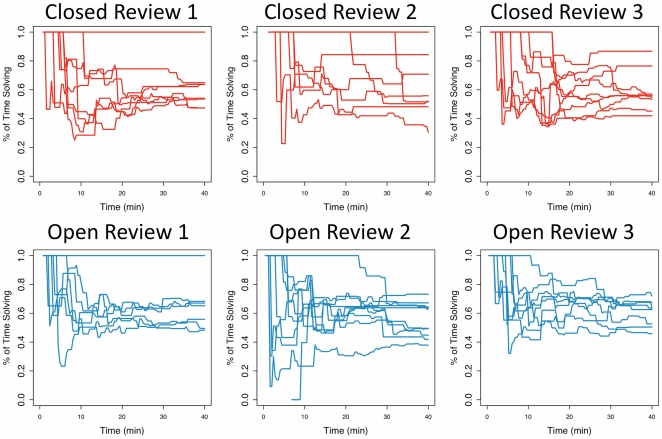
Open peer reviewers spend a greater proportion of their time reviewing. Each panel is a plot of the cumulative proportion of time each individual spends solving problems during the experiment over the course of the game. Under closed peer review, individuals spend a greater proportion of their time solving (top row). In two experiments (Closed Experiment 1, Closed Experiment 2), an individual spent almost 100% of their time solving problems. Under open peer review, individuals spent a smaller proportion of their time solving problems and a greater proportion of their time reviewing problems (bottom row).

Review times were not significantly different between open and closed review (2 seconds longer on average for closed games, P = 0.31), consistent with observations from randomized controlled trials [12]. However, in the closed games players spent a higher proportion of their time solving problems instead of reviewing (Figure 2 top row), while in the open games, there was a greater balance between reviewing and submission (Figure 2 bottom row). In two of the closed experiments, individuals spent nearly all of their time solving problems; this behavior was only observed once in the open experiments.

Finally, overall reviewing accuracy was statistically indistinguishable between open and closed peer review (1% more accuracy under closed, P = 0.762). This result agrees with previous studies of open and closed peer review which showed no statistically significant difference in review quality between the two systems [14].

#### Open review leads to increased cooperation which leads to increased review accuracy

**Figure 3:**
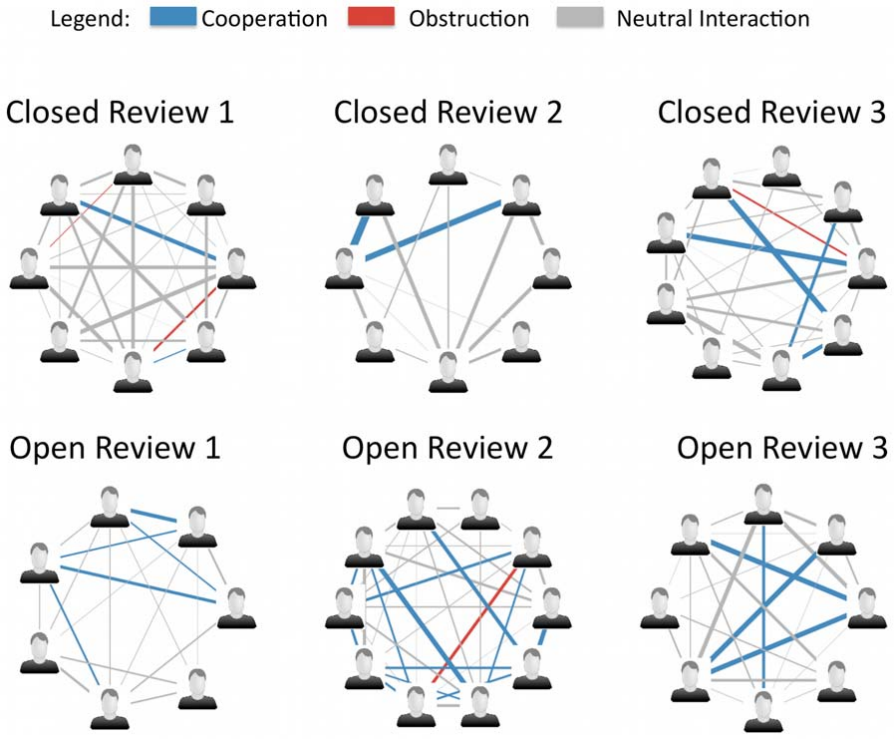
Open reviewers are more cooperative than closed reviewers. Each panel shows the cooperation network for one of the peer review experiments. The thickness of the line indicates the amount of interaction and color indicates the type of interaction. Cooperation (blue) is defined as above average probability of both players accepting each others solutions. Obstruction (red) is defined as below average probability of both players accepting each others solutions. Under closed review (top row) there is less cooperation between players than under open review (bottom row).

An important question is whether making reviewing behavior public facilitates cooperation. For each experiment we calculated a pair-wise measure of cooperation between players (Materials and Methods). The open review experiments showed more cooperative connections than the closed experiments (22% versus 9% respectively, P = 0.018, Figure 3). It was not immediately clear that cooperation between referees and authors would increase reviewing accuracy. Intuitively, one might expect that players who cooperate would always accept each others solutions - regardless of whether they were correct. However, we observed that when a submitter and reviewer acted cooperatively, reviewing accuracy actually increased by 11% (P = 0.016). The difference in accuracy was significant even after adjusting for the fact that some solvers had higher accuracy than others (11% increase in accuracy, P = 0.039). The increase in reviewing accuracy was mediated by cooperative interactions between players, since overall accuracy was comparable under open and closed peer review (1% more accuracy under closed, P = 0.762).

## Discussion

We have developed both a theoretical and experimental model for peer review. Our theoretical model allows exploration of the relative impact of alternative systems and incentives for peer review. A basic analysis of the theoretical model suggests that the current system of anonymous peer review discourages reviewing activities. Further exploration of the model under alternative systems and incentives may be helpful in evaluating alternative models of review going forward. Using our experimental model, we were able to collect the first data on social interactions and accuracy under alternative peer review models. Our experimental results both substantiate our theoretical model and agree with previous studies of peer review systems. We have also shown that one mechanism for increased cooperation is making reviewer information public. But other mechanisms for improving cooperation in the review process may exist; for example, reducing calls for unnecessary experiments has recently been suggested as a potential improvement in the reviewing process [21]. Our results indicate that improved cooperation does in fact lead to improved reviewing accuracy. These results suggest that in this era of increasing competition for publication and grants, cooperation is vital for accurate evaluation of scientific research.

## Materials and Methods

### The Peer Review Game

**Figure 4:**
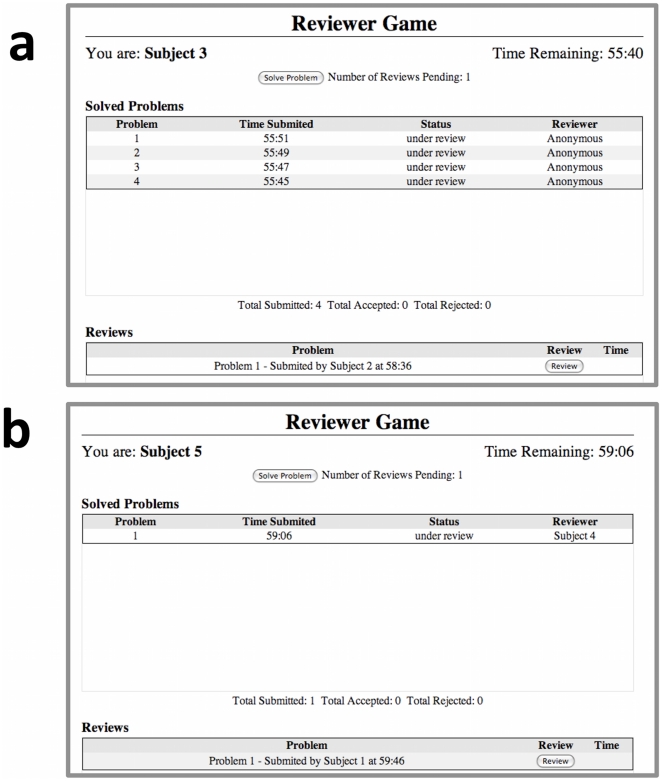
The task selection screens for the Peer Review Game. Task selection under the (**a**) closed and (**b**) open modes. In each case a player may elect to solve or review a problem. In the open peer review mode, players know the identity of the players reviewing their solutions.

**Figure 5:**
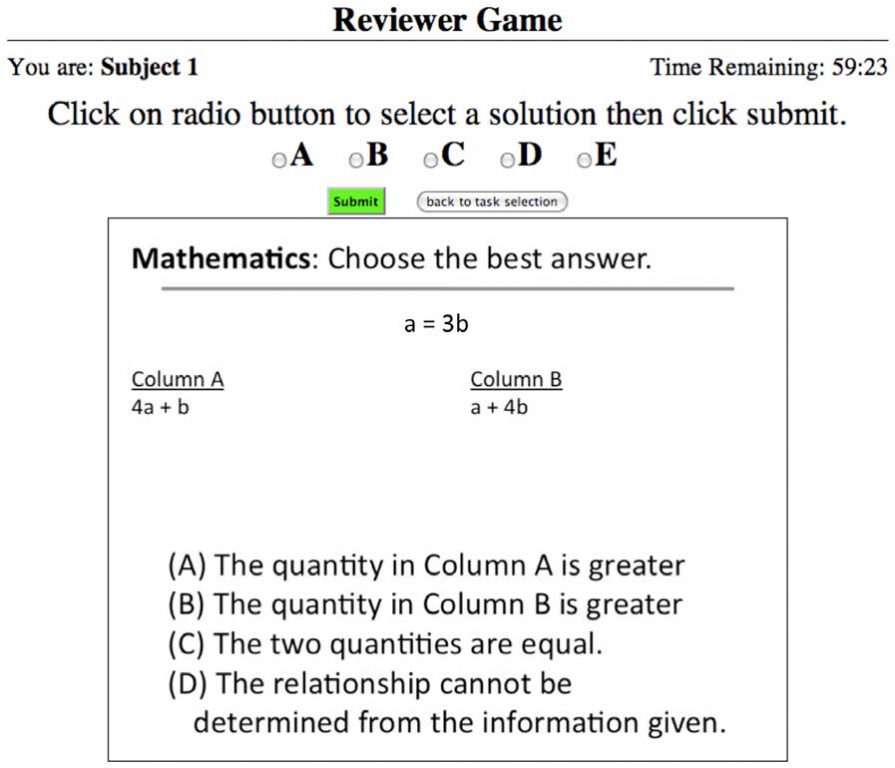
The problem solving screen for the Peer Review Game. The problem solving screen is the same for both versions of the game.

**Figure 6:**
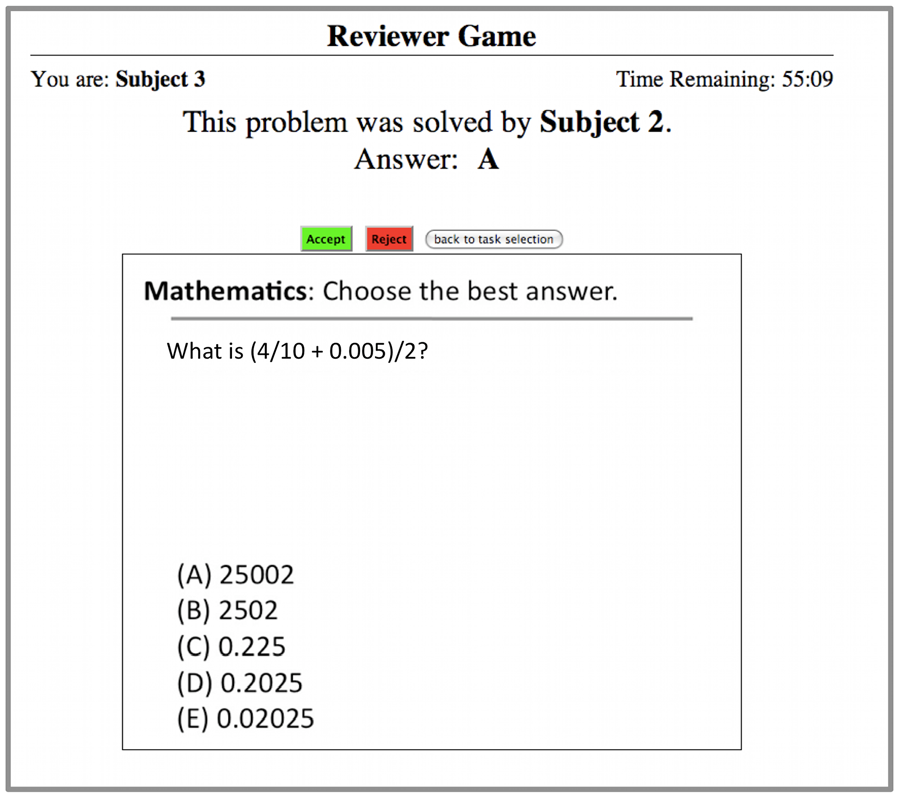
The reviewing screen for the Peer Review Game. The reviewing screen is the same for both versions of the game.

We developed a peer review game that can be played by two or more players. The game was developed as an Amazon Machine Image (AMI) that can be launched from the Amazon Elastic Compute Cloud [22]. The game was developed using the vWorker online development platform [23]. Players were directed to a website of a temporary web-server and logged on with a user name and password. When the investigator initiated the game, the players were shown a task selection page (Figure 4). They could choose to solve a problem or choose to review a problem from their list of pending reviews. If a player chose to solve a problem, then a GRE-like problem was selected from a database for them to solve and displayed to their screen (Figure 5). The GRE problems used for the experiment were based on problems from the website [19]. If they chose to review a problem, then they were shown a solution to a problem submitted by one of their peers (Figure 6). They could choose to either accept or reject the solution to the problem. The program acted as editor, randomly assigning problems to players for peer review.

**Figure 7:**
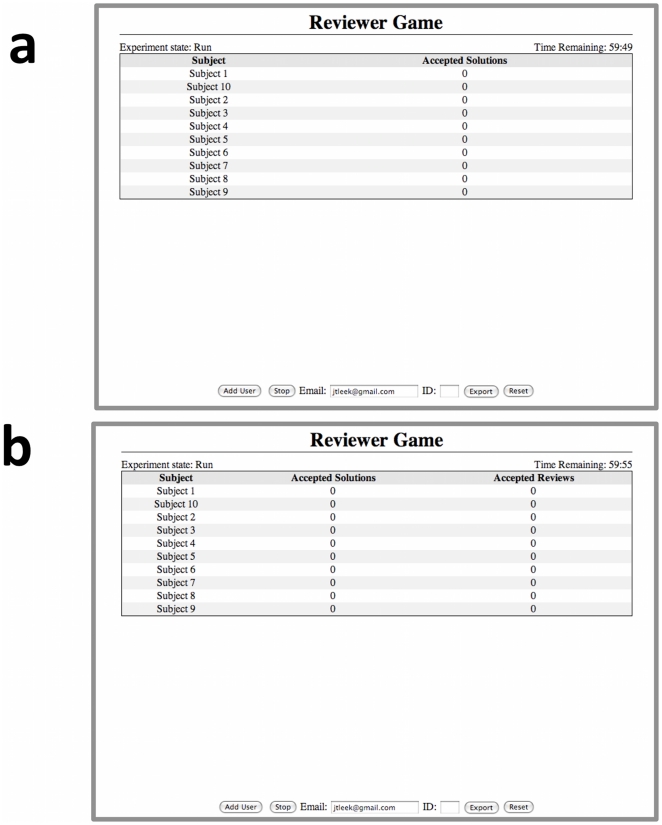
The public information screens for the Peer Review Game. Public information under the (**a**) closed and (**b**) open modes. In each case the number of solutions each player has had accepted are displayed. In the open review system, the number of solutions reviewed and accepted by each player is also displayed.

In both the open and closed games reviewers were shown the identity of the player who solved the problem. Under the open system, solvers were also shown the identity of the player who acted as peer reviewer for their solution. Throughout the game, information was projected onto a screen at the front of the room. In the closed mode, the number of solutions each player had submitted and had accepted was displayed (Figure 7a). In the open mode, the number of solutions each player had reviewed and accepted for one of their peers was also displayed (Figure 7b).

At the beginning of each game, the players were read the instructions for the appropriate mode (closed or open) as described in the following sections. The investigator then initiated a session of the Peer Review Game that lasted for T = 40 minutes in each case. Nametags were given to each subject with their anonymous subject ID at the beginning of the experiment and players were permitted to speak to one another during the course of the experiment.

### Recruitment

Six laboratories at the Johns Hopkins Medical School and Johns Hopkins Bloomberg School of Public Health were recruited to participate in the peer review experiment. Laboratories consisted of graduate students, postdoctoral fellows, research scientists, and principal investigators. Each laboratory participated in one replication of the Peer Review Game; the goal was to mimic the small and relatively tight-knit communities of scientists who act as peer reviewers for each other's papers. Experiments were performed on laboratories of laboratories of size K = 8, 8, and 9 players for the closed game and K = 7,10, and 8 players for the open game. Participating laboratories were offered $50 for each 10 participating members of the lab, a complimentary lunch, and the potential for two lab members to earn $5 each. Written informed consent was obtained from all participants in the study. Recruitment was performed with approval from the Johns Hopkins Bloomberg School of Public Health IRB, project number 3316.

### Group dynamics measurement

Next we estimated a measure of cooperation or obstruction between subjects 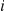 and 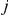. The baseline observed acceptance probability for subject 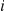, 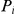 was calculated as 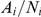 where 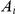 is the number of solutions accepted by subject 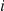 and 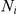 is the number of solutions reviewed by subject 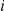. We computed the observed probability that subject 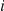 accepts a solution submitted by subject 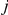, 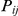, as 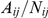, where 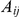 is the number of solutions accepted by subject 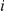 which were submitted by subject 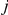, and 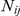 is the number of solutions reviewed by subject 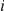 which were submitted by subject 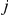. The difference 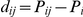 gives a measure of the change in the probability subject 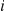 accepts a solution from subject 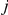 relative to their overall acceptance rate. Similarly, we can calculate 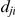 as a symmetric measurement. If 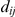 and 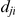 are both positive, then the interaction between the two subjects is cooperative. Similarly, if both values are negative, the interaction between the two subjects is obstructive. We calculated the total number of possible interactions under both the open and closed peer review experiments. Among these, we identified the number that were cooperative. We then performed a two-sample test of proportions to evaluate whether there was more cooperation under OPR or CPR.

### Outcome modeling

In all outcome modeling, the unit of observation is one reviewed problem. Each reviewed problem has a solver and a reviewer and is associated with a particular study type, either open or closed.

To control for differences in behavior between individual participants, the models described below were fit using a mixed-model framework, with all models including separate random effects for solvers and reviewers. Model fitting was done in the statistical programming language R [24] using the function glmer from the package lme4 with a linear link assuming Gaussian distribution of random effects [25]. In a general form the random effects model can be written as
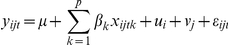
where 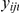 is the outcome of interest related to a review at time 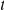 by subject 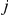, for a solution submitted by subject 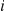; 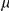 is the mean outcome over the whole data set; 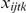 is the 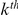 covariate of interest which has effect size 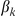, 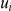 is a random effect associated with subject 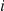 and 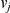 is a random effect associated with subject 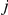. We assume that 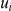, 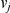 and 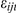 are mean zero Normal random variables with variances 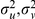 and 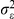, respectively.

To assess the impact of previous review performance by a subject on the chance that solutions submitted by that subject will be accepted, we associated to each reviewed problem the number of solutions accepted by the problem submitter, up to the time the problem was reviewed. In the open framework, this value was known to all study participants, including the reviewer; in the closed framework, this value was unknown.

Modeling the acceptance probability of a submission as a function of this covariate and the study type, and their interaction, we assessed the change in acceptance probability for each solution accepted by the submitter, in either the open or closed review setting. Define 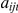 to be the indicator that solution 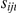 is accepted. The model is then:
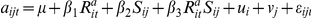
where 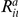 is the number of reviewed and accepted solutions by subject 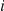 by time 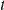, 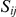 is an indicator of the study type that subjects 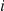 and 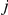 participated in (taking a value of 0 for closed review and 1 for open review). In this model 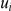 is a random effect representing the solver, 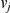 is a random effect representing the reviewer and 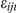 represents residual variation not due to reviewer or solver effects.

To assess the impact of the open or closed scenarios on review quality, we associated to each reviewed problem an indicator of whether the review was accurate, given that we know the correctness of the submitted solution.

We defined the variable 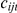 to be an indicator of whether solution 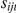 was correctly reviewed (e.g. accepted if correct, rejected if incorrect). To assess the impact of cooperation on review accuracy, for each reviewed problem, we defined a 0–1 indicator 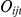 which takes a values of 1 if subjects 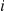 and 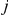 have a cooperative interaction. We then fit the model
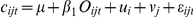
where all terms are as defined above.

To ensure the effect observed in this model is not due only to the increased accuracy of the solution submitted by the problem solver, for each reviewed problem we defined a three-level factor, with level 0 indicating that neither the solver nor the reviewer was part of a cooperative pair, 1 indicating that only the solver was part of a cooperative pair, and 2 indicating that both the solver and the reviewer are part of a cooperative pair. Calling this variable 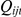 we then fit the model
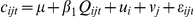
where all terms are as defined above.

We also modeled this accuracy as a function of study type alone to determine whether one scenario produced more accurate reviews. We fit the model
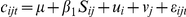
where all terms are as defined above.

### Instructions for the Closed Peer Review Games

#### Purpose of research project

This research is being done to evaluate open and closed peer review systems experimentally. Peer review is the process by which scientific research is evaluated for publication in journals. The goal of this study is to determine whether anonymous (closed) or non-anonymous (open) peer review results in more correct research being accepted.

#### Why you are being asked to participate

You are being asked to participate in the study because you are a graduate student, postdoctoral research fellow, scientist, or faculty member at Johns Hopkins University and are representative of the population of individuals who will participate in the peer review process.

#### Procedures

Once the experiment begins, you will be asked to answer multiple choice questions similar to questions on the graduate record exam (GRE). After you submit your answer, the solution will be randomly assigned to another participant in the study for review. The reviewer can either choose to accept or reject the solution. The reviewer will know your subject ID. However subjects who submit solutions will not know the ID of the reviewer of their solution. Throughout the course of the experiment you will act as both a reviewer and a problem solver. You may spend as much time as you like on either task. The experiment will last for forty minutes. I will now show you example screens from the experiment website and you may ask questions about the study procedure.

#### Risks/discomforts

You may experience some stress since you will be asked to answer GRE like problems and review the solutions of your peers. However, the only interaction you will have with other participants will be through the anonymous subject IDs.

#### Payment

The two individuals with the most accepted answers at the conclusion of the experiment will receive $5. The payment will be in cash immediately following the experiment. If you leave the study early you will lose your opportunity to win the cash prizes distributed at the end of the experiment.

#### Protecting data confidentiality

All research projects carry some risk that information about you may become known to people outside of a study. We minimize these risks by not connecting your responses to any information that could be used to identify you. All data collected during this experiment will only be connected with the anonymous subject ID you have been assigned.

#### Protecting subject privacy during data collection

Your responses and reviews will not be personally associated with you. All interaction will be performed based on the anonymous subject IDs you have been assigned.

#### What happens if you leave the study early?

You may leave the study at any time without penalty.

### Instructions for the Open Peer Review Games

#### Purpose of research project

This research is being done to test open and closed peer review systems experimentally. Peer review is the process by which scientific research is evaluated for publication in journals. The goal of this study is to determine whether anonymous (closed) or non-anonymous (open) peer review results in more correct research being accepted.

#### Why you are being asked to participate

You are being asked to participate in the study because you are a graduate student, postdoctoral research fellow, scientist, or faculty member at Johns Hopkins University and are representative of the population of individuals who will participate in the peer review process.

#### Procedures

Once the experiment begins, you will be asked to answer multiple choice questions similar to questions on the graduate record exam (GRE). After you submit your answer, the solution will be randomly assigned to another participant in the study for review. The reviewer can either choose to accept or reject the solution. The reviewer will know your subject ID and you will know the reviewer ID for each solution after it is reviewed. Throughout the course of the experiment you will act as both a reviewer and a problem solver. You may spend as much time as you like on either task. The experiment will last for one forty minutes. I will now show you example screens from the experiment website and you may ask questions about the study procedure.

#### Risks/discomforts

You may experience some stress since you will be asked to answer GRE like problems and review the solutions of your peers. However, the only interaction you will have with other participants will be through the anonymous subject IDs.

#### Payment

The two individuals with the most accepted answers at the conclusion of the experiment will receive $5. The payment will be in cash immediately following the experiment. If you leave the study early you will lose your opportunity to win the cash prizes distributed at the end of the experiment.

#### Protecting data confidentiality

All research projects carry some risk that information about you may become known to people outside of a study. We minimize these risks by not connecting your responses to any information that could be used to identify you. All data collected during this experiment will only be connected with the anonymous subject ID you have been assigned.

#### Protecting subject privacy during data collection

Your responses and reviews will not be personally associated with you. All interaction will be performed based on the anonymous subject IDs you have been assigned.

#### What happens if you leave the study early?

You may leave the study at any time without penalty.

### Reproducible Research

To conform with the standards of reproducible research, R [24] scripts and R data objects have been posted at: http://www.biostat.jhsph.edu/~jleek/peerreview/ that reproduce all analyses performed in this paper.

### Informed Consent

Written informed consent was obtained from all participants in this study. This specific study was approved by the Johns Hopkins Bloomberg School of Public Health IRB with project number 3316.
